# Co-locating frailty intervention activities and general practice: a qualitative study exploring experiences and perceptions of participants and delivery partners

**DOI:** 10.3399/BJGPO.2025.0007

**Published:** 2026-04-08

**Authors:** Alexandra Thompson, Luke Robertshaw, Barbara Hanratty, Michael Ali, Jennifer Liddle

**Affiliations:** 1 Population Health Sciences Institute, Newcastle University, Newcastle upon Tyne, UK; 2 Rise, North Shields, UK

**Keywords:** physical activity, frailty, ageing, exercise, primary health care, general practice, care pathways

## Abstract

**Background:**

Physical activity interventions targeting frailty improve health in later life. Integrating these interventions with primary care services may enhance perceptions and outcomes of delivery. Every Move Matters (EMM) is an 8-week group-based frailty intervention aimed at improving physical ability, social connection, and wellbeing. Recruitment is embedded within primary care pathways. EMM is delivered by external partners within or near general practice venues.

**Aim:**

To explore the experiences and perceptions of participants, intervention providers, and primary care staff involved in delivering the EMM frailty intervention, with a focus on the impact of its co-location within or near general practice settings.

**Design & setting:**

A rapid inductive qualitative evaluation of the EMM intervention co-located with primary care services.

**Method:**

Semi-structured interviews were conducted with nine EMM attendees, six primary care staff, and three staff members from the activity provider organisation between October 2023 and March 2024. Transcripts were analysed thematically.

**Results:**

Three themes were developed: straightforward implementation and adaptation; effectiveness; and buoyant atmosphere. EMM’s location within primary care reassured attendees and fostered enthusiasm and support from primary care staff, promoting recruitment and feelings of pride. A dedicated embedded postholder with access to patient records could enhance recruitment.

**Conclusion:**

Implementing co-located interventions in collaboration with primary care is possible and has benefits for intervention attendees, delivery staff, and primary care practitioners. The potential to embed other interventions within primary care settings could be explored. Closer collaboration with primary care may promote access to data for evaluation of the longer-term benefits and sustainability of a range of interventions.

## How this fits in

Physical activity interventions have shown promise in improving outcomes for those living with, or at risk of, frailty. However, such interventions are often located in community centres or other community settings. Little is known about any potential benefits or limitations of provision within or near to primary care settings. This study provides perspectives of attendees and delivery partners of an 8-week group-based frailty intervention, and indicates that such sites are ideally situated for the delivery of community-based frailty-focused activity interventions and that benefits can be experienced for all involved.

## Introduction

Global trends in population ageing reflect increases in life expectancy.^1^ However, healthy life expectancy has not increased, with up to half of people aged >85 years experiencing frailty. This state of increased physical and mental vulnerability involves low grip strength, exhaustion, slow walking pace, and unintentional weight loss,^2^ and is associated with poor health, multiple health conditions, falls, hospitalisation, long-term care placement, and mortality.^3^ Routine identification and management of frailty in older people is recommended in general practice in England.^4^

Physical activity can enhance mobility and independence in older adults by improving cognitive, physical, and psychological function. Multicomponent exercise programmes combining resistance, aerobic, balance, and flexibility training show promise in addressing frailty,^5^ although evidence on long-term benefits and broader implementation remains limited.

The NHS Long Term Plan and the establishment of primary care networks promote integration of care services tailored to local populations.^6^ This includes collaboration between primary care services and voluntary and community organisations. Co-locating services has been associated with better communication, collaborative working, and improved patient outcomes, as seen with mental health service integration in GP practices.^7,8^ Evidence also shows that co-location in community settings can reduce health inequalities, foster trust between providers and patients, and improve service coordination and acceptability.^9,10^ However, activity interventions co-located with primary care, particularly those delivered by external partners, have not, to our knowledge, been previously studied. Co-location may increase trust and uptake, especially for people at risk of frailty who often cite concerns about safety, falls, and health conditions as barriers to activity.^11^

Every Move Matters (EMM) is a community-based programme, embedded within routine primary care pathways, to support older people’s physical and social wellbeing. EMM is facilitated by Rise^12^ and delivered by local voluntary and community sector partners: Active Families North East (https://activefamiliesne.co.uk) and Gorilla Training (https://mailchi.mp/ff68de693007/gorillatraining). The programme targets older adults living with mild frailty in areas of greater deprivation, thus at greater risk for poor health outcomes. Potential attendees are identified by primary care teams through patient record searches. Text messages sent via primary care systems invite eligible individuals, and response to the message indicates consent to share data with the provider. Eligible participants are aged >55 years, live independently, and coded as mildly frail on primary care systems using the Electronic Frailty Index^13^ or Clinical Frailty Scale (scores 3–5; managing well to mildly frail). Activity providers then contact individuals with session details. See Figure 1 for the procedure for identification and recruitment to EMM.

**Figure 1.**
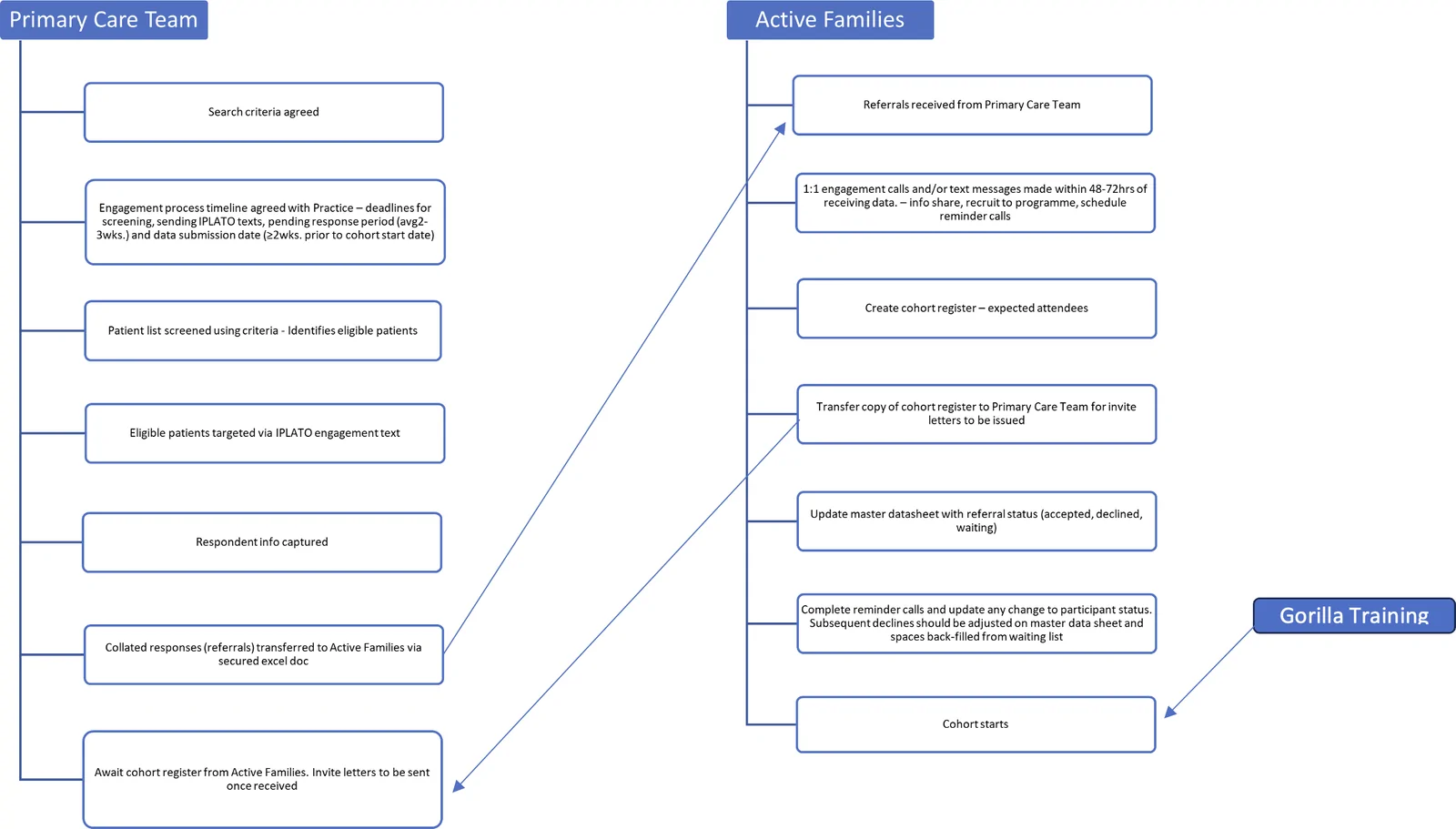
Procedure for identification and recruitment to the Every Move Matters programme. avg = average. hrs = hours. wks = weeks.

EMM consists of weekly, hour-long instructor-led sessions over 8 weeks. These include functional strength and cardiovascular exercises and activities such as bowls and movements with pom-poms. Educational sessions are grounded in life coaching and motivation, with some focused specifically on health and exercise. Social interaction is encouraged throughout, both during activities and over refreshments. A ‘Next steps’ maintenance programme with a similar activity format is offered to attendees deemed independent.

Originally a 16-week programme delivered at a charity-led community health centre in Newcastle upon Tyne, EMM was found to be acceptable and feasible in a rapid qualitative evaluation, with participants and staff reporting physical and mental health improvements.^14^ The programme was subsequently adapted based on these findings. Changes included providing a DVD to sustain home activity, reduced duration to improve attendance, tailored session content to match participant abilities and interests, and more structured integration of social and physical activities. Team-based and competitive elements were prioritised, and delivery protocols improved to support ongoing activity signposting.

EMM has since expanded to four additional sites across Tyne and Wear, with all sessions now co-located in or near general practice settings. To determine any benefit or impact of this co-location, this study explores the experiences and perspectives of attendees and collaborating staff involved in EMM’s implementation and delivery.

## Method

This study follows the Consolidated Criteria for Reporting Qualitative Research^15^ and was conducted from October 2023 to March 2024.

### Topic guide

Semi-structured interview guides were developed for each responder type in consultation with clinical experts and programme managers from Rise. A prior evaluation of the original 16-week programme, which included public input, informed the topic guide’s focus. This highlighted areas for further exploration such as recruitment, motivation, setting suitability, and sustainability.^14^ Topics were designed to elicit experiences and perceptions most relevant to each participant group (Figure 2).

**Figure 2.**
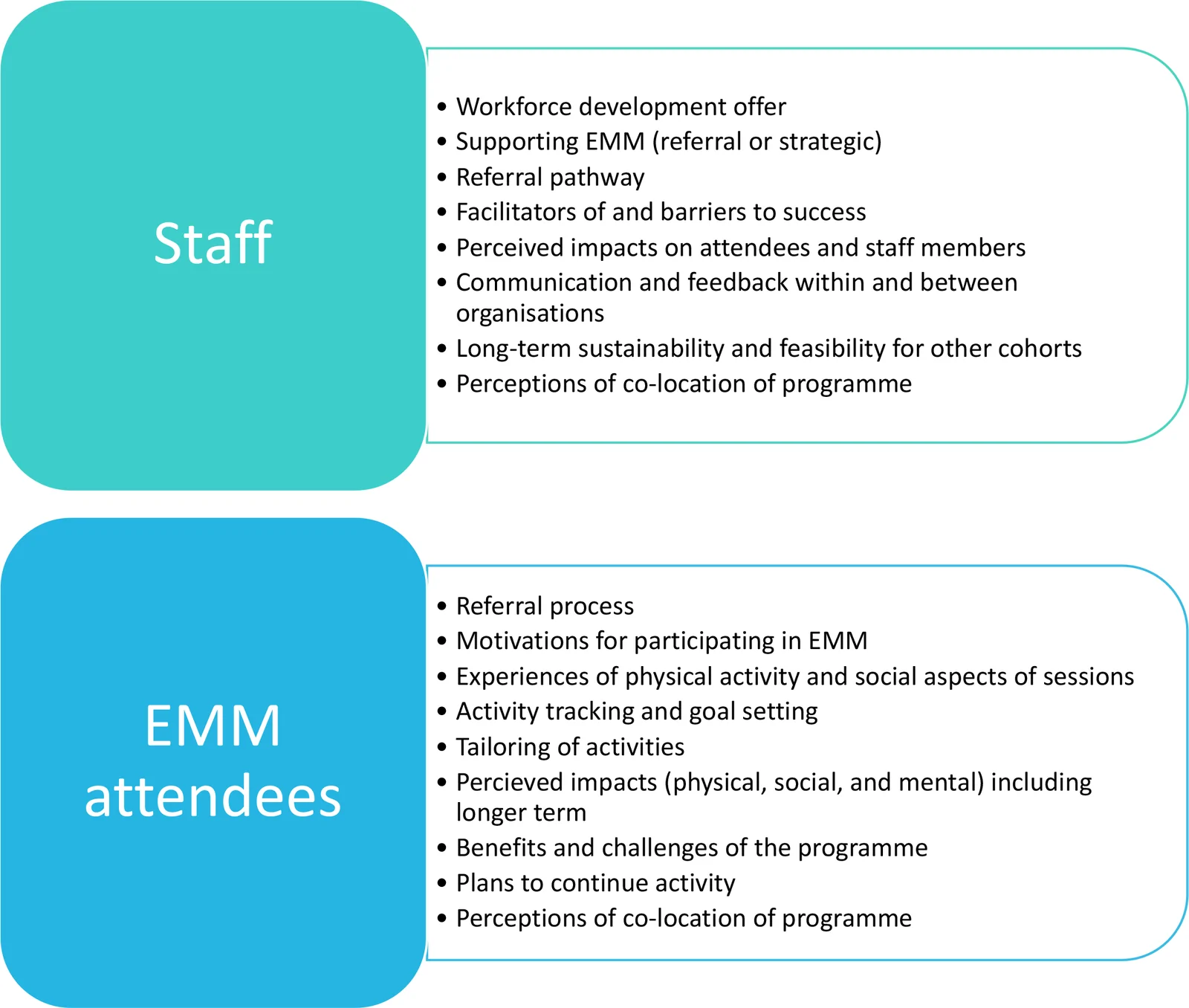
Every Move Matters (EMM) interview topics by responder type

### Participants and recruitment

Purposive sampling ensured representation from each delivery site and from both staff and attendee perspectives. Eligible attendees had completed the programme 1–3 months prior. Rise identified eligible staff and attendees based on their involvement and availability; the research team then made contact. Although the time lag supported reflection and potential identification of longer-term impacts, we acknowledge that interviews up to 3 months post-intervention may have been affected by recall bias.

Introductory emails were sent to prospective staff interviewees, with an attached information sheet. Attendees were contacted by email or telephone. Interested individuals received an information sheet (by email or post) and were then contacted to schedule an interview.

### Procedure

Before interview, introductions were made, procedures and aims explained, and informed consent was obtained. Interviews were conducted using Zoom, Microsoft Teams, telephone, and in person in private rooms in researchers’ or staff interviewees’ workplaces. Interviews were conducted by AT (female, research associate, 4 years’ qualitative research experience) and LR (male, public health specialist, trained in qualitative methods). No interviews were conducted in participants’ homes. Semi-structured interviews included open and focused questions, with prompts to explore key topics. Interviewees received a £25 shopping voucher in recognition of their time. Interviews were recorded, transcribed, and anonymised, with data managed according to UK General Data Protection Regulation and the Data Protection Act 2018. Immediately after each interview, researchers documented key impressions and reflections.

### Data analysis

Reflexive thematic analysis (RTA) was employed owing to its flexibility and focus on meaning-making from interviewees’ perspectives.^16^ This approach suited the study’s aim of exploring diverse experiences among healthcare staff, activity providers, and older adult participants. RTA enabled inductive pattern identification without a predefined coding framework and allowed reflexive engagement with researcher positionality.

The team met regularly to discuss emerging impressions and topics during data collection. Transcripts were inductively coded by AT using NVivo (version 14.23.2), software used to support analysis of qualitative data. Initial codes were organised in a matrix to identify patterns and key findings. Similar codes were grouped into themes, which were discussed and refined by AT and JL.

## Results

### Responder characteristics

Eighteen interviews were conducted: nine with EMM staff and nine with attendees from three of the four sites (Table 1). Attendees had a mean age of 72 years, all identified as White British, and 89% were female. Interviews lasted 39–55 minutes; three were conducted in person, one via Zoom, and five by telephone.

**Table 1.** EMM participant responder characteristics

Characteristic	EMM participants (*N* = 9), *n* (%)^a^
**Age, years, mean (range)**	72 (65–83)
**Sex**	
Male	1 (11)
Female	8 (89)
**Ethnicity**	
White British	9 (100)
**Marital status**	
Divorced	2 (22)
Married	7 (78)
**Housing tenure**	
Homeowner	2 (22)
Renter	7 (78)
**Living arrangements**	
Living alone	2 (22)
Living with partner	7 (78)
**Employment status**	
Retired	9 (100)
**EMM location**	
Newcastle	3 (33)
Gateshead	2 (22)
South Tyneside	4 (44)

^a^Unless otherwise stated. EMM = Every Move Matters.

Data on marital status, housing tenure, and living arrangements were collected for context but did not appear to influence participant experiences.

Nine EMM staff were interviewed: three from the external activity provider and six from the primary care network, representing all four sites (Table 2). Activity staff included two directors and one administrator; primary care staff included a clinical director, four managers, and a care team lead. All identified as female and were White British. Interviews lasted 21–33 minutes; seven were conducted online on Microsoft Teams, two in person.

**Table 2.** EMM staff responder characteristics

Characteristic	EMM staff (*N* = 9), *n* (%)^a^
**Age, years, mean (range)**	49 (38–67)
**Sex**	
Female	9 (100)
**Ethnicity**	
White British	9 (100)
**Employment status**	
Full-time employed	8 (89)
Part-time employed	1 (11)
**Role designation**	
Primary care	
Clinical director	1 (11)
Practice/service manager	4 (44)
Care team lead	1 (11)
Activity provider	
Directors	2 (22)
Administrator	1 (11)
**Years in profession or role, mean (range)**	6.2 (0.45–16)
**Organisation**	
Primary care	
Newcastle	1 (11)
South Tyneside	2 (22)
Gateshead	2 (22)
Washington	1 (11)
External activity provider	3 (33)

^a^Unless otherwise stated. EMM = Every Move Matters

### Interview themes

The following three themes were generated: straightforward implementation and adaptation; effectiveness; and buoyant atmosphere. Interview themes and illustrative quotes are presented below.

#### Straightforward implementation and adaptation

This theme relates to EMM’s implementation into primary care settings and related adaptations. No major implementation challenges were reported, and communication among primary care, the provider, and Rise was considered effective. Screening and referral processes exceeded expectations without adding to workload. GP-led recruitment was seen as more trustworthy than external outreach. Staff suggested broadening recruitment to include people seeking weight loss support or from marginalised groups. The lack of ethnic diversity among current cohorts was noted.

##### Primary care staff support and enthusiasm

Support from primary care staff was viewed as key to the programme’s success. Staff from across sites felt that investment from senior members and belief in the programme had been vital to implementation. Primary care collaborators had seen the value in EMM and had taken ownership of its realisation. There was a perception that this had resulted in improved uptake in the more supportive practices that used additional recruitment methods such as leaflets and social media. These positive experiences engendered a desire for continued collaboration in the future. One senior staff member described recognising the programme’s potential and choosing to support it:

*‘Sometimes you’ve just got to be able to see that little glimmer of a star of something that’s promising. So that was my sort of buy-in; I can see that this is something good.’* (Staff [S]4)

However, some sites appeared less invested, potentially affecting recruitment. Some senior staff also reported feeling distanced from day-to-day EMM activities and wanted increased feedback about progress of EMM. It was felt that increased access to patient records for EMM staff could aid the programme. This could potentially be via a dedicated staff member embedded within GP practices, physically, socially, and interpersonally, and/or with access to IT systems, with limited responsibilities beyond recruitment and coordination for the programme, which could improve programme uptake and foster a stronger sense of ownership within primary care teams.

Although the topic guide included questions about the workforce development offer, few interviewees raised this during interviews. Co-location did not appear to impact on training either positively or negatively. Staff from the activity provider did not mention participating in training, and primary care staff, mainly managerial, reported either being unaware of the offer or feeling they already had sufficient knowledge. The training offer therefore did not appear to have influenced implementation or delivery in these sites.

##### Location

EMM participants felt reassured by the proximity of EMM activity sessions to GP surgeries, valuing the sense of safety of having healthcare staff nearby and noting a perception that the activity was approved. However, this perception of safety was participant-led; healthcare professionals were not directly involved in EMM sessions and there were no formal arrangements for managing medical emergencies beyond standard procedures such as calling emergency services if needed:

*‘… the point that I said yes really, was ... the fact that it was … a doctor facility, you know, so if anything was to take wrong, you felt safe because the doctors were ... around the corner ... for the first time of doing something like this, after that sort of thing* [onset of epilepsy and associated loss of confidence] *happening to you … it was important to know that you were going to be safe.’* (Participant [P]6)

Where GP surgeries lacked space, nearby alternatives, such as community centres, were used. Activity provider staff emphasised the benefit of having suitable venues in close proximity to health centres, typically up to a 10-minute walk.

Venues were valued for their accessibility and welcoming atmosphere, which staff felt made participants feel more comfortable and confident. Venues were particularly appreciated when managers were supportive, flexible, and helped create a positive environment for attendees. The proximity of primary care locations was seen as enhancing primary care support for the programme’s delivery:

*‘I was in the office next to the session ... just to hear the laughter, hear the music and when I would be walking out I’d see like the patients ... I’d always have a chat with the patients to say how’s it going ... the feedback was so positive … that I just thought this has … been an amazing piece of work, amazing.’* (S2)

Interviewees valued having follow-on activities from the programme situated nearby to allow participants to gradually transition, building their confidence to take up activities independently.

Adaptations were needed during the EMM implementation in primary care settings. For instance, staff initially raised concerns about noise from sessions at one GP practice, but these were swiftly resolved. Staff noted that addressing these challenges helped strengthen relationships between the organisations involved:

*‘… the music was playing and the doctors were still having surgery ... So I went straight back ... and said, ‘we’ve had some feedback ... is there any way we can just start the sessions a bit earlier when the doctors ... aren’t in surgery?’ And straight away we adjusted it so we’ve developed a really good relationship between the three … organisations … any issues we’ve highlighted they’ve picked it up and dealt with.’* (S2)

##### Capacity

Both EMM participants and staff raised concerns about room capacity, noting that limited space could be an issue for future cohorts owing to growing interest in the programme. Staff felt that more people could benefit from EMM, but expansion was hindered by resource and venue limitations. While locating activities near GP practices was a priority, this restricted flexibility for new settings. However, smaller group sizes were seen as beneficial for promoting social interaction.

### Effectiveness

This theme highlights benefits of EMM for participants and staff. Many continued home exercises up to 3 months post-programme, reporting improved balance and mobility. Mood changes were harder to assess, particularly among those with mental health conditions.

#### Positive mindset

Participants reported increased positivity and motivation to lead full and active lives post-EMM attendance. They felt inspired by the diverse abilities and ages of attendees and intended to continue physical activity, even in less structured forms. Improved balance and awareness of limb movements increased confidence to venture outdoors, and the supportive environment, particularly encouragement from instructors, helped participants tackle new challenges. One participant gained the confidence to remove their hat during sessions, something they hadn’t done in public since surgery, and felt empowered to do so in other settings as well:

*‘I’ve got two bits of skin graft on, so I’ve got no hair on the top, and I always had a hat on, and we were just chatting one day, and she* [activity instructor] *said, “You always have your hat on ... If you want to take your hat off, you take it off. We all love you here.” ... “We respect whatever you want to do.” ... and I took my hat off, and I’ve taken it off in other places, as well. It was her that gave me the confidence. I still get a little bit emotional. And it was all talked about nice … comfortable.’* (P5)

The intervention fostered social connections, although some participants lacked the time or mental resources to maintain them. Many formed new friendships, which positively impacted psychological wellbeing. These groups often continued physical activity together and were a motivating factor for enrolling in the 'Next steps' maintenance programme after EMM.

#### Unexpected benefits

Unexpected benefits of the programme were noted for participants, staff, and the wider community. Some participants' family members joined sessions to offer support and ended up completing the programme themselves. While this was viewed positively by staff and participants, these additional attendees were not formally recruited using the same eligibility criteria, and their inclusion occasionally contributed to space constraints.

Staff from primary care settings expressed feelings of satisfaction, pride, and joy from their involvement. Hearing participant laughter and receiving direct or indirect feedback, particularly from socially isolated patients, was felt to have a positive impact on staff:

*‘... it’s the highlight of my day … it just makes your day when you hear the difference it makes to people lives, you know. It’s lovely ... I feel a little bit kind of like emotional about it because they come out with phrases like, “It’s the best thing in my life at the moment.”’* (S11)

#### Proactive approach

The EMM recruitment approach was seen as crucial. Participants viewed the text and telephone-call invitations as timely prompts to make changes they had been considering. The invitations were seen as non-intrusive, a *‘gentle push’* (P6), with some participants interpreting the timing as a *‘sign’* (P9) owing to personal circumstances such as illness or bereavement. The involvement of healthcare professionals added reassurance and legitimacy to the programme:

*‘… it was from my GP surgery, which made me feel 100% better about it … ’* (P7)

Some participants actively sought information about the programme at their general practice, sometimes after picking up a leaflet advert. Referral staff and participants emphasised that social prescribers accompanying individuals to sessions boosted confidence and could be done more easily in such co-located venues.

### Buoyant atmosphere

This theme highlights the positive atmosphere created by EMM. Participants consistently described the sessions as uplifting and mood-boosting, with a warm and friendly environment fostered by both attendees and staff.

#### Nostalgia

Nostalgia was a common theme, especially regarding the choice of music, which participants associated with their era. The music promoted interaction, laughter, increased energy, and improved mood. The tailored approach, responsive to participants' feedback, was appreciated, with elements such as skipping ropes evoking memories from earlier life:

*‘... it was not just the exercises, it was the music that the instructors picked … was from our era … when we were younger … I was doing exercises thinking, “Oh, I could get up and have a dance here,” you know, like a proper dance … And I thought that was great … everybody was singing along … Everyone was smiling.’* (P5)

#### Novelty

Novelty also played a key role. Participants enjoyed trying new activities, such as movements with pom-poms and drumsticks, which they found motivational and fun:

*‘... she came in with drumsticks and the vast majority of people were so excited about these drumsticks. So that was obviously passed on, and brought them in a couple of times ... people who thoroughly enjoyed that did it.’* (P9)

Despite minimal initial hesitation, most found the activities enjoyable and motivational, although one participant noted that the light-hearted nature might not appeal to everyone:

*‘I saw one person that I knew and I asked. They were like, “Oh, it wasn’t for me.” And I was like, “Well why?”* [and they said] *“I don’t know, it just wasn’t for me.” They didn’t enjoy it, but there’s nothing wrong with the classes, it just wasn’t their style and they didn’t like it.’* (P3)

#### Uplifting activity staff

Participants held highly positive views of EMM staff, describing them as warm, friendly, and caring. Staff were seen as key in motivating and creating enjoyable sessions, with participants appreciating the rapport built, such as being greeted by name and asked about their wellbeing:

*‘... like I say, the* [health education session leader] .*.. made me think, “Yes, I’ve got to think about myself.” ... honestly, I mean, I’m not very good with my memory, but she was a good impact on people, she was ... The instructors were lovely.’* (P5)

Staff encouragement had notable impacts, helping participants to view situations differently and create a safe, confidence-building environment. Participants also appreciated the attention to injuries and pre-existing conditions. However, primary care staff were unclear about who should manage discussions on participants' suitability, suggesting that the activity provider should handle these decisions after enrolment.

## Discussion

### Summary

This rapid qualitative evaluation explored a physical and social activity intervention for individuals with or at risk of mild frailty, co-located with primary care. Proximity to a healthcare setting reassured participants of its appropriateness and facilitated quick issue resolution, strengthening support and recruitment. Participants reported increased activity, confidence, motivation, and social connection. Staff and family members also benefited, with staff noting improved wellbeing. The programme’s success was attributed to its uplifting atmosphere, supportive instructors, and a blend of nostalgia and novelty.

### Strengths and limitations

This study provides insights from primary care staff, delivery partners, and EMM participants. While attendees from three of four sites were interviewed, staff from all four and the delivery partner contributed. Interviews conducted up to 3 months post-programme allowed some exploration of longer-term impacts.

As a qualitative study with a small sample, it was not designed to assess variation across sociodemographic factors such as ethnicity, sex, or socioeconomic status. Most staff and participants were female and White British. Limited ethnic diversity, previously noted in EMM’s first evaluation, persisted.^14^ Future research with larger, more diverse samples and mixed methods could explore these dimensions further.

Many participants had joined the 'Next steps' programme, which shared staff, locations, and activities; therefore, it was difficult to disentangle their experiences of EMM from those of the maintenance programme. All participants had engaged with EMM, so views are likely positively biased. Co-location may not suit all sites or individuals; some may feel intimidated by proximity to healthcare providers, especially those with prior negative experiences of complex health systems.^17^

Although the topic guide included prompts on areas such as activity tracking and participant challenges, these were not strongly reflected in responses. This may suggest they were not central to participants’ experiences, or other aspects were more salient.

To date, EMM has been assessed primarily through qualitative methods focused on acceptability and feasibility. No formal quantitative or cost-effectiveness evaluations have been conducted. Future studies should incorporate quantitative outcomes and economic analyses to assess broader impact and sustainability.

### Comparison with existing literature

Interviewees noted that co-locating EMM with primary care reassured frail or at-risk individuals, addressing known activity concerns about safety and falls.^18^ This appears to be the first report highlighting the significance of proximity to medical support in activity interventions. Our findings align with emerging evidence that co-location of health and social services can enhance collaboration, build trust, reduce health inequalities, and improve accessibility and acceptability.^9,10^ These benefits support the potential for co-located frailty interventions, such as EMM, to promote uptake and sustained engagement.

Attendees also felt reassured by GP-linked invitations, which lent legitimacy to the referral. While encouragement from healthcare professionals is known to support uptake of activity schemes, and community-based delivery is often preferred over hospital venues, the influence of referral legitimacy appears to be a novel finding.^19,20^

Primary care staff felt co-location fostered ownership, pride, and positive morale, enhancing recruitment and implementation. Activity provider staff suggested that improved access to patient records, or embedding the programme more fully within primary care, could strengthen recruitment by increasing provider engagement and shared responsibility.

Evidence indicates that physical activity interventions delivered in primary care settings, in collaboration with other interventionalists, such as exercise experts, are effective at increasing activity in individuals at risk of disease or who live with long-term health conditions.^21^ However, there is little evidence examining the benefits to staff in these settings, the ease of implementation of interventions into such locations, or perspectives of participants of this format.

Co-locating EMM near primary care settings supported its implementation. In implementation theory, successful implementation depends on social mechanisms driven by individual contributions leading to collective action.^22^ Key elements include capacity, potential, capability, and contribution. Locating EMM near GP practices increased capacity and potential,^22^ facilitated resource mobilisation, cooperation, and coordination, while fostering individual and shared commitments between collaborating organisations. Despite the ease of implementation, implementing in other sites and settings may prove more challenging owing to workforce pressures on primary care services. Developing a shared vision with mutual benefits may be crucial for the successful implementation of similar programmes in co-located settings.

The positive atmosphere throughout the intervention considerably impacted on the psychological wellbeing of attendees. This atmosphere included encouragement of social interaction throughout the activity sessions, which could account for the formation of social connections being more evident in the current iteration of EMM. However, this uplifting environment was largely attributed to EMM staff; seen as crucial in increasing motivation, confidence, mood, and fostering a safe, goal-oriented space. Staff contribution,^22^ a key factor in implementation theory, was essential to the programme’s success. This atmosphere also influenced primary care staff and attendees' families. Most attendees continued to the 'Next steps' maintenance programme, citing the uplifting environment as a key motivator.

Research supports that activity instructors who are attentive, caring, enthusiastic, and create warmth contribute to the success of similar programmes.^23,24^ Enjoyment and fun are also critical for the acceptability and effectiveness of exercise interventions for older adults.^25–27^ Prioritising these aspects in programme design can improve engagement and outcomes, while strengthening collaboration between primary care and intervention teams.

The popularity of EMM led to the perception of new challenges for future cohorts. Staff members and programme participants noted that limitations in existing resources, such as staff numbers and venue space, created uncertainty about how the capacity of the programme could be increased given the demand.

Although much evidence suggests that practical factors such as transport links and accessibility are important for activity intervention success,^24,28^ there is little knowledge relating to the activity space itself or that concerns the resourcing of programmes. As some responders highlighted the potential benefits of smaller group sizes, simply finding a larger activity space may not be the answer.

Both EMM attendees and staff deemed the current programme format sustainable. Staff highlighted the team’s capability^22^ to integrate EMM into existing workflows, supporting its feasibility. However, staff noted that the 8-week programme alone may be insufficient to promote lasting behaviour change, aligning with existing literature.^29^ The inclusion of a ‘Next steps’ exit route was considered essential. Evidence suggests that maintenance interventions enhance the effectiveness of similar programmes.^11^ Staff emphasised the importance of familiar settings to encourage participation, supported by enthusiastic attendee feedback. Prior research underscores the role of confidence-building and promoting local exercise opportunities in fostering long-term physical activity.^5^

### Implications for research and practice

The reported benefits of co-location on uptake and adherence to the EMM intervention, as well as for primary care staff, suggest this approach is successful (Figure 3). Implementing co-located delivery for similar interventions may enhance attendees' activity levels, social connections, as well as psychological wellbeing in all parties.

**Figure 3.**
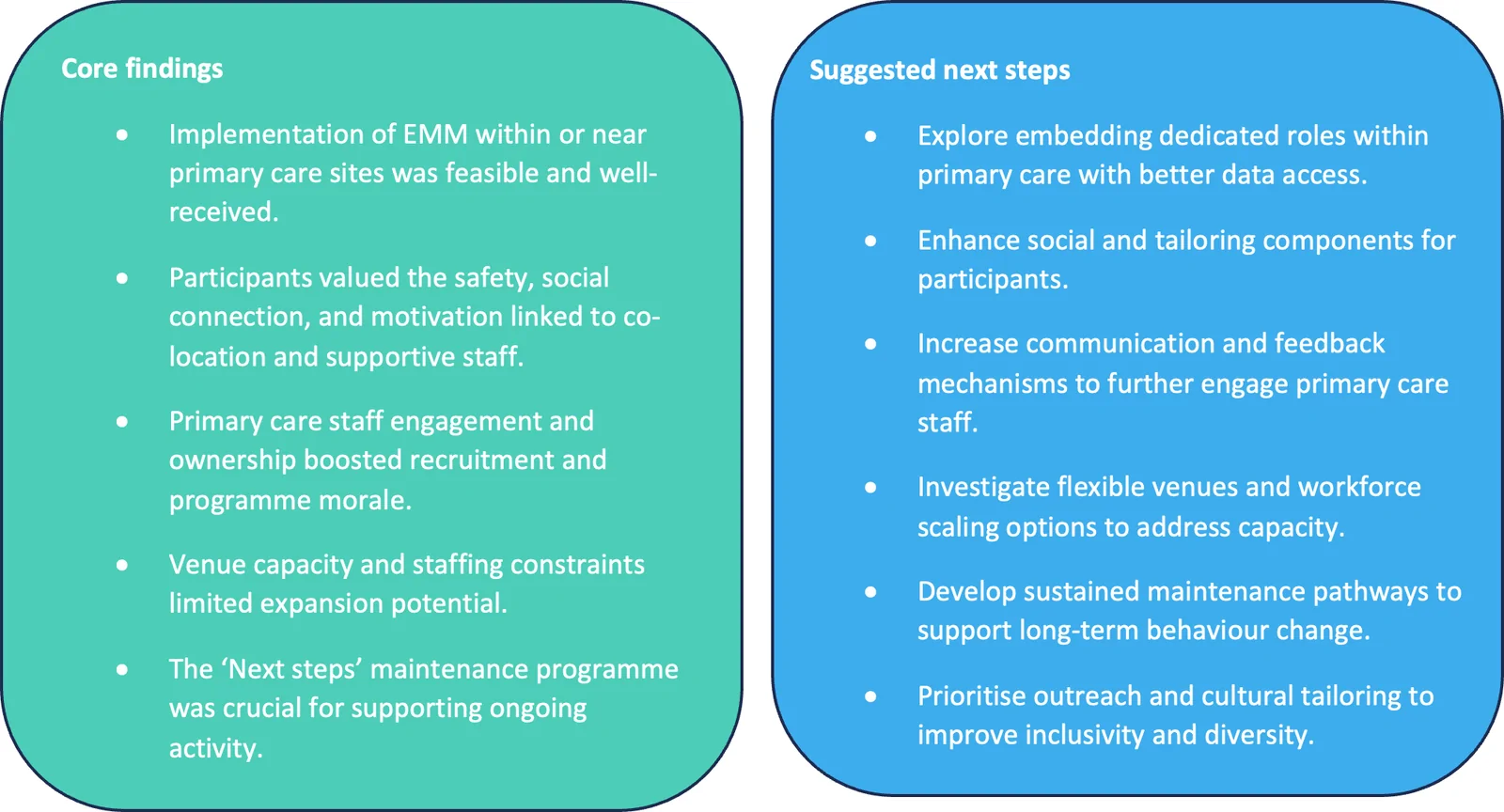
Core findings and next steps

Embedding external activity intervention partners within primary care settings could strengthen these collaborations. This may improve access to patient records, promote proactive recruitment, and provide health data to demonstrate benefits, supporting long-term sustainability.

The role of intervention delivery staff is crucial. In our intervention, staff possess the right skills to positively impact attendees and primary care staff. Recruiting appropriately skilled personnel is essential for the success of similar programmes.

To maximise impact, balancing growth with efficacy and acceptability is key. Ensuring sustainability of programmes while addressing resourcing issues is critical, especially as provisions can be discontinued or reach capacity. A flexible approach, such as repurposing primary care or nearby spaces, may be beneficial, as would designing new practices with community spaces for intervention delivery.
